# Algorithm for the treatment of type 2 diabetes: a position statement of Brazilian Diabetes Society

**DOI:** 10.1186/1758-5996-2-35

**Published:** 2010-06-08

**Authors:** Antonio C Lerario, Antonio R Chacra, Augusto Pimazoni-Netto, Domingos Malerbi, Jorge L Gross, José EP Oliveira, Marilia B Gomes, Raul D Santos, Reine MC Fonseca, Roberto Betti, Roberto Raduan

**Affiliations:** 1 Medicine School of Universidade São Paulo, InCor - HCFMUSP Diabetes Core. Sao Paulo, Brazil; 2 Paulista Medicine School at Universidade Federal São Paulo - UNIFESP, Sao Paulo, Brazil; 3 Integrated Center of Cardiovascular Hypertension and Metabology at the Kidney and Hypertension Hospital at Universidade Federal de São Paulo, São Paulo, Brazil; 4 Diabetes Center of Hospital Alemão Oswaldo Cruz, in Sao Paulo, Brazil; 5 University of Sao Paulo Medical School. Sao Paulo, Brazil; 6 Medical School at Universidade Federal Rio Grande do Sul and Hospital Clínicas in Porto Alegre, Porto Alegre, Brazil; 7 Medicine School of Universidade Federal Rio de Janeiro and Division of Nutrology and Diabetes Service at UFRJ Hospital Universitário Clementino Fraga Filho, Rio de Janeiro, Brazil; 8 Medicine School of State University of Rio de Janeiro, Rio de Janeiro, Brazil; 9 InCor Lipid Clinical InCor - HCFMUSP, Sao Paulo, Brazil; 10 Universidade Federal da Bahia - UFBA, Salvador, Brazil; 11 Diabetes Core. InCor- HCFMUSP and Diabetes Center of Hospital Alemão Oswaldo Cruz, Sao Paulo, Brazil; 12 Internal Medicine Service at Beneficencia Portuguesa, Sao Paulo, Brazil

## Abstract

The Brazilian Diabetes Society is starting an innovative project of quantitative assessment of medical arguments of and implementing a new way of elaborating SBD Position Statements. The final aim of this particular project is to propose a new Brazilian algorithm for the treatment of type 2 diabetes, based on the opinions of endocrinologists surveyed from a poll conducted on the *Brazilian Diabetes Society *website regarding the latest algorithm proposed by *American Diabetes Association */*European Association for the Study of Diabetes*, published in January 2009.

An additional source used, as a basis for the new algorithm, was to assess the acceptability of controversial arguments published in international literature, through a panel of renowned Brazilian specialists. Thirty controversial arguments in diabetes have been selected with their respective references, where each argument was assessed and scored according to its acceptability level and personal conviction of each member of the evaluation panel.

This methodology was adapted using a similar approach to the one adopted in the recent position statement by the American College of Cardiology on coronary revascularization, of which not only cardiologists took part, but also specialists of other related areas.

## Module 1

### Summary of Brazilian Diabets Society Members Opinions on the New ADA/EASD Algorithm

Considering the great controversy raised by the recommendations at the recent ADA/EASD algorithm, the Brazilian Diabetes Society (BDS) decided to evaluate the opinions of its members, through a survey conducted on the BDS' website during ten days, in November 2008 [1,2]. Two hundred and seventeen associates (endocrinologists) completed this survey.

Table 1 shows the percentages of answers to the proposed questions to BDS' associates.

**Table 1:** Percentuals of answers to the proposed questions

	ANSWERES' PERCENTAGES		
	
PROPOSED QUESTIONS	Yes	No	Others
*1. Have you read the Treatment of Type 2 Diabetes algorithm proposed by ADA/EASD?*	90	10	-
2. Were you aware that the document expresses the opinion of a few authors and not of the entities involved?	77	23	-
3. Do you intend to adopt the stages and steps suggested by this algorithm in your practice?	51	49	-
4. Do you think that rosiglitazone-associated adverse events have been ratified in the medical literature of excellence?	36	64	-
5. Do you think that the cardiovascular protection assigned topioglitazone in that Position Statement is real?	34	66	-
6. Do you think that glitazone-associated adverse events (bone fracture and cardiovascular events) are effects pertaining to this therapeutical class?	49	21	30

7. Do you think that only GLP-1 analogs should be included in diabetes treatment excluding DPP-4 inhibitors?	13	69	18
8. Do you think that BDS' members have the expertise and the ability of criticism to issue a Position Statement about this Algorithm?	87	4	9

Gomes MB. *Enquete sobre Algoritmo ADA/EASD - December 2008 - 217 Sócios da SBD*. Available at: http://www.diabetes.org.br/agenda/comunicados/index.php?id=1838. Accessed on: July 13, 2009.

#### General conclusions about the survey results

The results showed that the majority of the brazilian endocrinologists do not agree with the guidelines proposed by the ADA/EASD algorithm regarding the use of glitazone, GLP-1 analogs and DPP-IV inhibitors in the treatment of Type 2 Diabetes.

Considering the need for an algorithm reflecting the opinion of Brazilian endocrinologists, the Brazilian Diabetes Society decided to develop this position statement, whose recommendations shall be dictated by the technical panel assessments, named by the entity and also by the results obtained from the survey.

## Module 2

### Results of the controversial diabetes argument acceptability assessment

In addition to the feedback from associates obtained through the survey and in order to provide a more robust basis to the algorithm proposed for the treatment of type 2 diabetes, the Brazilian Diabetes Society obtained the opinions of a panel formed by renowned Brazilian specialists regarding recommendations, guidelines and controversial arguments on the treatment of type 2 diabetes in international literature.

Thirty controversial arguments were individually assessed and scored on a 10-point scale by the evaluation panel members, who assigned individual scores (0-10) to the 30 arguments presented, which were made into 5 acceptability levels (1-5).

The correlation between scores and their corresponding acceptability levels, as well as the analytical interpretation of results, are summarized in table 2.

**Table 2:** Interpretation of acceptability levels of arguments based on individual scores

Score	Acceptability Level	Interpretation
0 - 2	1	Full rejection
3 - 4	2	Partial rejection
5 - 6	3	Neutrality
7 - 8	4	Partial acceptance
9 - 10	5	Full acceptance

#### Average acceptability level of controversial matters assessed and their respective bibliographical references

Table S1, Additional file 1 shows the relation between controversial matters assessed and their respective bibliographical references and the average level of acceptability for each controversial matter, following the calculation methodology as defined in the previous item.

## Module 3

### New SBD algorithm proposal for the treatment of type 2 diabetes

#### Laboratory goals for characterization of good glycemic control

The desirable goal for A1C, as defined by the previous position statement in 2007, recommended A1C levels < 6.5%. In this new Position Statement, the recommended A1C goal was redefined to <7.0% as shown in table 3. However, according to the ADA's 2010 statement, in patients with a history of severe hypoglycemia, patients with limited life expectancies, children, individuals with comorbidities, those with longstanding diabetes, advanced age and those with advanced microvascular or macrovascular complications" intensive glycemic control may outweigh its benefits. But for patients with short duration of diabetes, long life expectancy, and no significant CVD a level of A1c even lower than the general goal of <7%, has been suggested if this can be achieved without significant hypoglycemia or other adverse effects [3-5].

**Table 3:** Laboratory targets for proper T2DM treatment

Parameter	Laboratory Targets	
	
	Desirable Levels	Tolerable Levels
Glycated hemoglobin (A1C)	<7% (in adults)	7.5-8.5%: 0-6 years old1; <8%: 6-12 years old1; <7.5%: 13-19 years old1; 8%: in the elderly^1,3^
Fasting glycemia	<110 mg/dL	Up to 130 mg/dL2
Pre-prandial glycemia	<110 mg/dL	Up to 130 mg/dL2
Post-prandial glycemia	<140 mg/dL	Up to 180 mg/dL2

Regarding tolerable levels for laboratory goals, they were defined based on the recommendations contained in the bibliographical references [3-5].

#### New SBD algorithm proposal for treating type 2 diabetes

*The new algorithm proposal for the treatment of type 2 diabetes was developed based on the premises and assessments conducted through the survey with SBD members and the assessment of conclusions from the panel of specialists *(Table 4).

**Table 4:** Algorithm for the treatment of type 2 diabetes - 2009 update -

STAGE 1: INITIAL CONDUCT ACCORDING TO CURRENT CLINICAL CONDITION			
**Mild manifestations**	**Moderate manifestations**	**Severe manifestations**	**Hospitalization if glycemic levels >300 mg/dL**

↓	↓	↓	↓

• Glycemic levels <200 mg/dL + • Mild symptoms or no symptoms + • Absence of other acute concomitant diseases	• Any glycemic levels between 200-300 mg/dL + • Absence of criteria for mild or severe manifestations	• Any glycemic levels above 300 mg/dL = Or = • Significant weight loss = Or = • Severe and significant symptoms = Or = • Presence of ketonuria	Under the following conditions: • Diabetic ketoacidosis and hyperosmolar state = Or = • Intercurrent severe disease or comorbidity

↓	↓	↓	↓

Metformin (500 mg/day, intensifying up to 2,000 mg/day) + lifestyle changes. If patient does not reach A1C<7% in 4-6 weeks → Note: In case of metformin intolerance, prolonged action formulations may be useful. If the problem persists, choose one of the options in Step 2	Metformin (500 mg/day, intensifying up to 2,000 mg/day) + lifestyle changes + other oral antidiabetic drugs CRITERIA FOR INCLUDING SECOND OAD ↓	Start insulin therapy immediately	Start therapy according to the algorithm recommendations and to glycemic control obtained after discharge from hospital

STAGE 2: ADD OR MODIFY SECOND AGENT ACCORDING TO A1C LEVEL(*)			

**7- 8%**	**8-10%**	**>10%**	

Sulphonylurea DPP-4 inhibitors Glitazone Glinides (prevalent post-prandial hyperglycemia) Acarbose (prevalent post-prandial hyperglycemia) Exenatide (overweight or obesity)	Sulphonylurea DPP-4 inhibitors Glitazone Basal insulin (bedtime) Exenatide (overweight or obesity)	Insulin therapy Basal insulin + prandial insulin With of without Metformin Sulphonylurea iDPP-4 (studies currently being made)	

(*) In order to select the second agent, we suggest looking at therapeutic drug profiles in table 7.			

**MONITORING AND ADJUSTMENTS IN TREATMENT AFTER 2-3 MONTHS WITH MAXIMUM EFFECTIVE DOSAGE IN ORDER TO REACH GOALS: A1C<7%, FASTING GLYCEMIA <130 mg/dL OR POST-PRANDIAL GLYCEMIA (2 HOURS) <180 mg/dL**			

**STAGE 3: ADD A THIRD ORAL AGENT OR INTENSIFY INSULIN TREATMENT**			

↓		↓	

Add a third oral agent with a different action mechanism. If in 2 or 3 months the targets of A1C<7%, fasting glycemia <130 mg/dL or post-prandial glycemia (2 hours) <180 mg/dL are not reached, start insulin therapy. →		Intensify insulin therapy until the A1C<7%, fasting glycemia<130 mg/dL or post-prandial glycemia (2 hours) <180 mg/dL goals are reached.	

INSTRUCTIONS AND ADDITIONAL COMMENTS			
1. Similarly to any other Guideline, this Algorithm contains general recommendations about the most highly indicated therapeutic options for each clinical situation. The choice of the best therapeutical plan must be made based on medical judgment, in patient's options and in treatment costs with the respective drugs.			
2. For further information on the potential of A1C level reduction of different drugs, please refer to table 6, in Module 4.			
3. For further summarized information on therapeutic and usage safety profile of several drugs, please refer to table 7, in Module 4.			

Abbreviations:			
A1C = glycated hemoglobin; inhibitors ofDPP-4 (dipeptidyl peptidase-4 ); OAD = oral antidiabetic drugs.			

The presentation format of the new algorithm proposal was developed taking as fundamental reference the recommendations by the Joslin Diabetes Center & Joslin Clinic and also by the American Association of Clinical Endocrinologists [6-9]..

The present algorithm was completed before the publication of the recent AACE/ACE algorithm [10]. As pointed out in the recent AACE algorithm safety, efficacy and effectiveness must be the priorities and in developing countries like Brazil cost of medications is an important barrier and could Influence the treatment.

## Module 4

### Summary of therapeutic profile of drugs used for treating type 2 diabetes

#### Comparative efficacy and potential of A1C reduction of different therapeutic interventions

The various therapeutic interventions present different levels of comparative efficacy and of potential of A1C reduction. Such facts must be taken into account when determining the best therapeutic strategy for each patient (table 5) [11,12].

**Table 5:** Comparative efficacy of therapeutic interventions for reducing A1C levels

Strategy/Drug	Expected Reduction in A1C (%)
Weight reduction and increase in physical activity	1.0 - 2.0
Metformin	1.0 - 2.0
Insulin as additional therapy	1.5 - 3.5
Sulfonylurea	1.0 - 2.0
Glitazones	0.5 - 1.4
GLP1 Agonists	0.5 - 1.0
DPP-4 Inhibitors	0.5 - 0.8
Alpha-glycosidase Inhibitors	0.5 - 0.8
Glinides	0.5 - 1.5

#### Summary of therapeutic profiles of drugs used for treating type 2 diabetes

The main features of therapeutic profiles of drugs used for treating type 2 diabetes are summarized in table 6[13,14].

**Table 6:** Pharmacologic options for oral DM-2 treatment

DRUG	PROFILE AND ACTION MECHANISM
Acarbose (Glucobay^®^)	Slows down intestinal glucose absorption. Low potential of A1C reduction (0.5 - 0.8%). Gastrointestinal intolerance.
Metformin (Glifage^®^, others)	Reduces primarily the hepatic glucose production and fights insulin resistance. High potential of A1C reduction (2%). Gastrointestinal intolerance. Does not cause hypoglycemia. May promote mild weight loss. Contraindicated in case of renal dysfunction.
Glitazones - Rosiglitazone (Avandia^®^) - Pioglitazone (Actos^®^)	Primarily fight insulin resistance and reduces hepatic glucose production. Increases muscle, fatty tissue and liver sensitivity to insulin. Intermediate A1C reduction potential (0.5 - 1.4%). Promote hydric retention and weight gain, increasing the risk of heart failure. Also increase the risk of fracture. Recent results of studies such as RECORD and BARI 2D indicate that rosiglitazone does not increase the risk of infarction and CVD.
Sulfonylureas - Glimepiride (Amaryl^®^) - Glibenclamide (Daonil^®^) - Gliclazide (Diamicron MR^®^) - Others	Stimulate endogenous insulin production by pancreatic beta cells, with pharmacological action medium to long (8-24 hours). Useful to control fasting glycemia and 24-hour glycemia. High potential of A1C reduction (2%). May cause hypoglycemia. Glibenclamide has higher risk of hypoglycemia. An alleged deleterious action on human beta cells has not yet been confirmed.
Glinides - Repaglinide (Novonorm^®^, Prandin^®^) - Nateglinide (Starlix^®^)	Stimulate endogenous insulin production by pancreatic beta cells, with short duration (1-3 hours). Useful to control post-prandial hyperglycemia. Intermediate potential of A1C reduction (1.0 - 1.5%). May promote weight gain and hypoglycemia. Repaglinide is more powerful than nateglinide.
Incretin mimetics and DPP-4 inhibitors - Exenatide (Byetta^®^) - Vildagliptin (Galvus^®^) - Sitagliptin (Januvia^®^)	This is a new therapeutic class for treating diabetes, whose mechanism includes stimulating beta cells to increase insulin synthesis and action on pancreatic alpha cells, reducing glucagon production. Glucagon has the effect of increasing glycemic levels. Average potential of A1C reduction (0.5 - 0.8%, depending on the basal A1C value). Do not cause hypoglycemia but gastrointestinal intolerance and pancreatitis have been described (exenatide and sitaglipitn) [13,14].

This table represents only a partial relation of commercial medications of various drugs and does not represent a specific recommendation of any commercial brand.

#### Fixed combinations of oral antidiabetic drugs

Due to its convenience and comparatively lower prices, fixed-combination therapies for treating diabetes are being made available more frequently. There are many presentations of combined treatments, including two oral agents in the same package, however with separate pills (table 7) or a single pill containing both active agents in the same formulation (table 8).

**Table 7:** Partial list of oral antidiabetic drugs used in combination therapy: two substances in separate pills

Therapeutic classes	Chemical Denomination	Commercial Denomination	Action and Dosage Mechanism
sulffonylurea + biguanide	glimepiride + metformin	Amaryl Flex^®^ Sanofi-Aventis	Long acting secretagogue of insulin (glimepiride) + peripheral action insulin sensitizer (metformin). Dosage: glimepiride - 1 mg and 2 mg + metformin - 500 mg.
glinide + biguanide	nateglinide + metformin	Starform^®^ Novartis	Short-acting secretagogue of insulin (nateglinide) + peripheral action insulin sensitizer (metformin). Dosage: nateglinide - 120 mg + metformin - 500 mg and 850 mg.

This table presents only a partial list of commercial medications of various drugs and does not represent a specific recommendation of any commercial brand.

**Table 8:** Partial relation of oral antidiabetic drugs at combination therapy: two substances in a single pill

Therapeutic classes	Chemical Denomination	Commercial Denomination	Action and Dosage Mechanism
biguanide + sulphonylurea	metformin + glibenclamide	Glucovance^®^ Merck	Peripheral action insulin sensitizer (metformin) + long-acting secretagogue of insulin (glibenclamide). Dosage: 250 mg metformin+1.25 mg glibenclamide 500 mg metformin + 2.5 mg glibenclamide 500 mg metformin + 5 mg glibenclamide.
glitazone + biguanides	rosiglitazone + metformin	Avandamet^®^ Glaxo	Combination of two peripheral action insulin sensitizers, with different action Dosage: 2 mg rosiglitazone + 500 mg metformin 4 mg rosiglitazone + 500 mg metformin.
Incretin mimetic + metformin	sitagliptin + metformin	Janumet^®^ MSD	DPP-4inhibitor + peripheral action insulin sensitizer (metformin). Dosage: 50 mg sitagliptin + 500, 850 or 1,000 mg metformin
Incretin mimetic + metformin	vildagliptin + metformin	Galvus Met^®^ Novartis	DPP-4 inhibitor + peripheral action insulin sensitizer (metformin). Dosage: 50 mg vildagliptin + 500, 850 or 1,000 mg metformin

This table presents only a partial list of commercial medications of various drugs and does not represent a specific recommendation of any commercial brand.

#### Action profile of human insulin and human insulin analogs

Basically, there are three commercial presentation forms of insulin in the Brazilian marketplace: 1) human insulin in monotherapy; 2) human insulin analog in monotherapy; 3) biphasic human insulin analogs.

The addition of insulin to patients with type 2 diabetes must be done as soon as the patient did not reach the target of HbA1c [15]. No definitve conclusions regarding the association between insulin therapy with glargine [16] and malignancies were established.

Table 9 summarizes the main features of the action profile of insulin preparations available.

**Table 9:** action profile of human insulin and human insulin analogs

Human Insulins	Insulin Type	Onset	Action peak	Duration of action
**Rapid-acting insulin analogs**	Glulisine (Apidra^®^)	<5-15 minutes	1 hour	4 hours
	Lispro (Humalog^®^)	<15 minutes	0.5-1.5 hour	2-4 hours
	Aspart (NovoRapid^®^)	5-10 minutes	1-3 hours	3-5 hours
**Short acting insulin**	Regular (Novolin^® ^R, Humulin^® ^R)	30-60 minutes	2-3 hours	3-6 hours
**Intermediate acting insulin**	NPH (Novolin^® ^N Humulin^® ^N)	2-4 hours	4-10 hours	10-16 hours
**Long-acting insulin analogs**	Glargine (Lantus^®^)	1-2 hours	None	Up to 24 hours
	Detemir (Levemir^®^)	1-2 hours	None	Up to 24 hours

This table presents only a partial list of commercial medications of various drugs and does not represent a specific recommendation of any commercial brand.

Biphasic insulin analogs have a long-acting insulin component, in a formulation combined with a short-acting insulin component, as shown in table 10

**Table 10:** Biphasic rapid and long-acting insulin analogs

Insulin aspart and protaminated (70%) + insulin aspart (30%)	NovoMix^® ^70/30	Pre-mix with 70% long-acting insulin aspart (up to 24 hours) + 30% aspart insulin of immediate release, short acting (4-6 hours), to control post-prandial and interprandial glycemic levels
Neutral protamine insulin lispro (75%) + insulin lispro (25%)	Humalog^® ^Mix 25	Pre-mix with 75% intermediate acting insulin NPL (up to 24 hours) + 25% insulin of immediate release, short-acting (4-5 hours), to control post-prandial and interprandial glycemic levels
Neutral protamine insulin lispro (50%) + insulin lispro (50%)	Humalog^® ^Mix 50	Pre-mix with 50% intermediate acting insulin NPL (up to 24 hours) + 50% insulin of immediate release, short-acting (4-5 hours), to control post-prandial and interprandial glycemic levels

This table presents only a partial list of commercial medications of various drugs and does not represent a specific recommendation of any commercial brand.

## Module 5

### Treatment cost estimate for various therapeutic options

The concept of Evidence-Based Medicine recognizes three main components to help physicians define therapeutic conduct: the evidence of research *per se*, the clinical expertise of physicians and patient preferences. Treatment cost must be one of the fundamental factors for patients to fulfill their right of choice in due proportion, in the concept of evidence-based medicine [17].

We added two website suggestions for physicians to obtain information about drug costs for consumers of the therapeutic options they intend to prescribe. In both references, prices are displayed in different rows expressing the costs of each drug, considering the incidence of distinct tax rates, which vary according to Brazilian states. For the physician, the desired piece of information is the maximum retail price (MRP), which may be found in the last row to the right in price tables.

### Links to browse drug prices

http://portal2.saude.gov.br/BPS/visao/consultapublica/publico_interno_item.cfm[18]

Using the internal resource of research to obtain the desired price http://www.elomedico.com.br[19]

Requires previous and free subscription. Search for item "drugs/prices" on the left row in the homepage

## Competing interests

ACL - The author declare that he has no competing interest

ARC - Research grants from Eli Lilly, Sanofi-Aventis, Roche, Novartis, MSD, Novo Nordisk

Advisory board: AZ, BMS, Sanofi-Aventis, Novo Nordisk

APN - External Medical Consultant for Roche Diagnostics; Speaker GSK, Eli Lily

DM - Committee Member: NONE

Research Grant: NONE

Speakers' Bureau: SANOFI-AVENTIS

Honoraria: SANOFI-AVENTIS

Expert Witness: NONE

Stock Ownership: NONE

Board of Directors: NONE

Consultant:: NONE

Scientific Advisory Board: SANOFI-AVENTIS

Steering Committee: NONE

JLG - Research Grants: Abbott, Bristol-Myers Squibb, Eli Lilly, Glaxo Smith Kline, Merck Sharp & Dohme, Novo Nordisk, Pfizer, Roche, Sanofi-Aventis

Speaker: Bristol-Myers Squibb, Eli Lilly, Glaxo Smith Kline, Merck Sharp & Dohme, Novo Nordisk, Sanofi-Aventis

Member Advisory Board: Glaxo Smith Kline, Merck Sharp & Dohme, Novo Nordisk, Sanofi-Aventis

JEPO - Member Advisory Board Sanofi-Aventis

MBG - The author declare that she has no competing interest

RDS - Member Advisory Board GSK, MerckSharp&Dhome

Speaker: Astra Zeneca, Pfizer, MerckSharp&Dhome, Bristol Meyers Squibb, GSK, Novartis

RMCF - The author declare that she has no competing interest

RB - The author declare that he has no competing interest

RR - Member Advisoy board Lantus

Speaker: SanofiAventis, Abbott, MerckSharp&Dhome, Eli Lily, Novonordisk, GSK

## Authors' contributions

APN wrote the manuscript.

MBG partipated in the design of the study and wrote the manuscript.

ACL, ARC, DM, JLG, JEPO, RDS, RMCF, RB, RR: all have partipated in the technical panel.

All authors read and approved the final manuscript.

## Supplementary Material

Additional file 1 **Table S1**. average acceptability level of controversial matters assessed and their respective bibliographic references. Click here for file
