# Supplementary material for: Algorithm for the treatment of type 2 diabetes: a position statement of Brazilian Diabetes Society
Source: Diabetol Metab Syndr. 2010 Jun 8;2:35. doi: 10.1186/1758-5996-2-35 (PMC2904721; doi:10.1186/1758-5996-2-35)
Supplement: Additional file 1 — Table S1. average acceptability level of controversial matters assessed and their respective bibliographic references. [file 1758-5996-2-35-S1.DOC]

**Table S1 – AVERAGE ACCEPTABILITY LEVEL OF CONTROVERSIAL MATTERS
ASSESSED AND THEIR RESPECTIVE BIBLIOGRAPHIC REFERENCES**

| **CONTROVERSY Nº** | **REFERENCES** | | **AVERAGE**  **ACCEPTABILITY**  **LEVEL** | | | |
| --- | --- | --- | --- | --- | --- | --- |
| **1 – *In the ACCORD study, intensive glycemic control caused a 23.6% increase in mortality rates as compared to the standard control group.*** | Action to Control Cardiovascular Risk in Diabetes Study Group. Effects of Intensive Glucose Lowering in Type 2 Diabetes. **N Engl J Med 2008**,358:2545-2559,  2008. | | **4** | | | |
| **2 – *In the ADVANCE study, intensive glycemic control significantly reduced the frequency of microvascular complications, but it did not cause a significant reduction in macrovascular outcomes or in cardiovascular events (MI, CVA and cardiovascular death).*** | ADVANCE Collaborative Group. *Intensive Blood Glucose Control and Vascular Outcomes in Patients With Type 2* *Diabetes*. **N Engl J Med 2008**,358:2560-2572, | | **4** | | | |
| **3 – *In the VADT study, intensive glycemic control did not significantly reduce the incidence of cardiovascular complications, as compared to the standard control group.*** | Glucose Control and Vascular Complications in Veterans with Type 2 Diabetes. **N Engl J Med** **2009**,360:129-139. | | **4** | | | |
| **4 – *In the DCCT-EDIC study assessing the long-term effects of intensive glycemic control in DM1 patients, nine years after DCCT was concluded, intensive glycemic control showed a 42% reduction in cardiovascular events and a 57% reduction in the risk of MI, CVA or cardiovascular death.*** | *Nathan DM.Intensive diabetes treatment and cardiovascular disease in patients with type 1 diabetes*. **N Engl J Med** **2005**, 353:2643-2653. | | **4** | | | |
| **6 – *A1C levels <7% reduce the risk of microvascular and neuropathic complications in DM1 and T2D. On the other hand, more recent clinical studies show conflicting results at cardiovascular risk reduction. In light of such conflict, it is recommended that the A1C<7% goal be maintained until new evidence becomes available.*** | | *Syler J.Intensive glycemic control and the prevention of cardiovascular events: implications of the ACCORD, ADVANCE, and VA Diabetes Trials. A position statement of the American Diabetes Association and a scientific statement of the American College of Cardiology Foundation and the American* *Heart Association*. **Diabetes Care 2009,**32:187-192. | | **4** | |  |
| **7 – *Glitazones reduce bone formation and accelerate bone loss in healthy, insulin-resistant individuals, increasing the risk of fracture in T2DM women.*** | | Grey A. *Skeletal consequences of thiazolidinedione therapy*. **Osteoporosis Int. 2008**,19(2):129-37.  *Meir C.Use of thiazolidinediones and fracture risk*. **Arch Intern Med 2008**,168:820- | | **4** | |  |
| **8 – *Rosiglitazone was associated with a significant increase in the risk of myocardial infarction and in conterminous significance in death from cardiovascular causes.*** | | Niessen SE, Wolski K.Effect of rosiglitazone on the risck of myocardial infarction and death from cardiovascular causes. **N Engl J Med 2007,**356:2457-2471. | | **2** | |  |
| **9 – *Pioglitazone was associated with a significantly lower risk of death, myocardial infarction and stroke among a diversified population of diabetes patients. Pioglitazone increased the risk of severe heart failure, with no associated mortality risk.*** | | *Lincoff AM et al.Pioglitazone and risk of cardiovascular events in patients with type* *2 diabetes mellitus. A meta-analysis of randomized trials*. **JAMA 2007,**298(10):1180-1188. | | **3** | |  |
| **10 – *Pioglitazone was associated with a 16% reduction in the cases of death, myocardial infarction and stroke, a controversial secondary outcome, which showed only marginal statistical significance.*** | | *Nathan DM et al .Medical management of hyperglycemia in type 2 diabetes: a consensus algorithm for the initiation and adjustment of therapy. A consensus statement of the American Diabetes Association and the European Association for the Study of Diabetes*. **Diabetes Care** **2008**,31:1-11.  *Yki-JarvinenH.The PROactive study: some answers, many questions***. Lancet 2005,**366(9493):1241-2. | | **3** | |  |
| **11 – *Pioglitazone shows beneficial effects and rosiglitazone shows neutral effect on atherogenic lipidic profiles.*** | | *Nathan DM et al .Medical management of hyperglycemia in type 2 diabetes: a consensus algorithm for the initiation and adjustment of therapy. A consensus statement of the American Diabetes Association and the European Association for the Study of Diabetes*. **Diabetes Care** **2008**,31:1-11.  Goldberg RB et al. *A comparison of lipid and glycemic effects of pioglitazone and rosiglitazone in patients with type 2 diabetes and dyslipedemia*. **Diabetes Care 2005,**28:1547-1554, | | **3** | |  |
| **12 – *Available data on cardiovascular risk with the use of rosiglitazone and on the cardiovascular benefits of pioglitazone are less than conclusive.*** | | *Nathan DM et al .Medical management of hyperglycemia in type 2 diabetes: a consensus algorithm for the initiation and adjustment of therapy. A consensus statement of the American Diabetes Association and the European Association for the Study of Diabetes*. **Diabetes Care** **2008**,31:1-11.  Dormandy JA et al. *Secondary prevention of macrovascular events in patients with type 2 diabetes in the PROactive Study (PROspective pioglitAzone Clinical Trial In macroVascular Events): a randomised controlled trial.* **Lancet 2005,**366:1279-1285. | | **3** | |  |
| **13 – *Even though the metanalyses considered are not conclusive with regards to the potential rosiglitazone-associated cardiovascular risk, and considering that other options are currently recommended, consensus group members are, unanimously, against the use of rosiglitazone.*** | | *Nathan DM et al.Medical management of hyperglycemia in type 2 diabetes: a consensus algorithm for the initiation and adjustment of therapy. A consensus statement of the American Diabetes Association and the European Association for the Study of Diabetes*. **Diabetes Care** **2008**,31:1-11. | | **1** | |  |
| **14 – *Glitazones improve insulin secretion capacity, reduce beta cell apoptosis and the amyloid contents of islets, while neogenesis is maintained.*** | | Wajchenberg BL.*Beta-cell failure in diabetes and preservation by clinical treatment*. **Endocr Rev.2007**, 28(2):187-218.  *A Diabetes Outcome Progression Trial (ADOPT)*. **Diabetes Care 2002,**25:1737-1743. | | **4** | |  |
| 15 – ***In clinical studies conducted up to now, DPP-IV inhibitors reduced A1C levels by 0.6-0.9 percentage points, with weight neutrality and relatively good tolerance, not causing hypoglycemia when used as monotherapy.*** | | *Nathan DM et al.Medical management of hyperglycemia in type 2 diabetes: a consensus algorithm for the initiation and adjustment of therapy. A consensus statement of the American Diabetes Association and the European Association for the Study of Diabetes*. **Diabetes Care** **2008**,31:1-11.  *Efficacy and Safety of Incretin Therapy in Type 2 Diabetes*. ***JAMA2007,*** 298:194-206. | | **4** | |  |
| **16 – *It is difficult to estimate the protective effects of incretin mimetics on beta cells in human beings and there is no clinical evidence that such drugs really bring protective effects to beta cells.*** | | Wajchenberg BL. *Beta-cell failure in diabetes and preservation by clinical treatment*. **Endocr Rev**. **2007**,28(2):187-218.  Meir JJ. *Beta cell mass in diabetes: a realistic therapeutic target*. **Diabetologia** 51:703-713, 2008. | | **4** | |  |
| **17 – *Anti-hyperglycemia agents with different action mechanisms have the highest synergy. Using insulin and metformin in combination is a particularly effective way of reducing glucose levels and limiting weight gain.*** | | Nathan DM. et al. *Medical management of hyperglycemia in type 2 diabetes: a consensus algorithm for the initiation and adjustment of therapy. A consensus statement of the American Diabetes Association and the European Association for the Study of Diabetes*. **Diabetes Care**  31:1-11, 2008.  Yki-Jarvinen. *Comparison of bedtime insulin regimens in patients with type 2 diabetes mellitus.* **Ann Int Med** 130:389-396,1999 | | **4** | |  |
| 18 – ***After starting therapy with metformin and life style changes in T2D, the addition of a second pharmacological agent must be made if A1C ≥ 7%.*** | | Nathan DM. et al. *Medical management of hyperglycemia in type 2 diabetes: a consensus algorithm for the initiation and adjustment of therapy. A consensus statement of the American Diabetes Association and the European Association for the Study of Diabetes*. **Diabetes Care 2008,**31:1-11.  Weng J. et al. *Effect of intensive insulin therapy on beta-cell function and glycaemic control in patients with newly diagnosed type 2 diabetes: a multicentre randomised parallel-group trial.* **Lancet 2008,**371:1753-1760. | | | **3** | |
| 19 – ***Starting or intensifying insulin therapy in patients with A1C levels A1C>8.5% or A1c >9.0% with symptoms secondary to hyperglycemia is indicated*** | | *Rodbard HW et al . Statement by an American Association of Clinical Endocrinologists/American College of Endocrinology Consensus Panel on type 2* diabetes mellitus:an algorithm for glycemic control.**Endocrine Practice** 2009;15:540-559. | | | **4** | |
| 20 – ***Prolonged exposure of human islets to different sulfonylureas caused different changes in the beta cell function, and glimepiride showed milder effects when compared to clorpropamide and glibenclamide.*** | | Del Guerra S. et al. *Effects of prolonged in vitro exposure to sulphonylureas on the function and survival of human islets*. **J Diabetes Complications 2005,**19(1):60-64. | | | **3** | |
| 21 – ***In general, using insulin was associated with increased risk of myocardial infarction. Such risk was higher in patients undergoing longer insulin therapy or who were concomitantly using sulphonylureas or biguanide.*** | | Margolis D.J. et al. *Association between serious ischemic cardiac outcomes and medications used to treat diabetes*. **Pharmacoepidemiol Drug Saf 2008,**17(8):753-9. | | | **2** | |
| 22 ***Type 2 diabetes insulin treatment was associated with reduced risk of cardiovascular events as compared to other treatments or with the absence of pharmacological treatment. In the group treated with insulin, the risk of CVD was reduced by 25% in patients under 65 years old and by 42% in patients between 31-45 years old.*** | | Engel-Nitz, N.M. et al. *Cardiovascular events and insulin therapy: a retrospective cohort analysis.* **Diabetes Res Clin Pract 2008,** 81(1):97-104. | | | **3** | |
| 23 – ***Long and shot acting insulin analogs offer few benefits as compared to conventional insulin in terms of glycemic control and reduction in hypoglycemic events.*** | | Singh SR et al. Canadian Agency for Drugs and Technologies in Health. *Efficacy and Safety of Insulin Analogues for the Management of Diabetes Mellitus: a Meta-Analysis*. **CMAJ 2009,**180(4):369-80. | | | **2** | |
| 24 – ***MI risk was higher among users of long-acting sulphonylureas (OR 2.07) than among users of new sulphonylureas (OR = 1. 36), as compared to OR’s among oral non-sulphonylureic antidiabetic drugs (1.38), of insulin (2.56) or in diabetes patients with no pharmacological treatment (3.51).*** | | Johnsen SP et al. *Risk and Short-Term Prognosis of Myocardial Infarction Among Users of Antidiabetic Drugs*. **Am J Ther 2006,**13(2):134-140. | | | **3** | |
| 25 – ***The treatment with sibutramine is associated with improvement in insulin sensitivity and with reduction in A1C levels in T2DM patients. In most studies, sibutramine caused favorable effects on lipids, particularly on HDL cholesterol and triglyceride levels, as well as on the total cholesterol/HDL cholesterol coefficient.*** | | Filippatos TD et al. *A Review of the Metabolic Effects of Sibutramine*. **Curr Med Res Opin 2005,**21(3):457-468.  Vettor R et al. *Effect of Sibutramine on Weight Management and Metabolic Control in Type 2 Diabetes: a Meta- Analysis of Clinical Studies*. **Diabetes Care 2005,**28(4):942-9.  Maggioni AP et al. *Tolerability of Sibutramine During a 6-Week Treatment Period in High-Risk Patients With Cardiovascular Disease and/or Diabetes: a Preliminary Analysis of the Sibutramine Cardiovascular Outcomes (SCOUT) Press release*  ** The Scout Study showed an increase risk of cardiovascular events in high risk patients (Early Communication about Ongoing Safety Review of Sibutramine,Food and Drug Administration*  *Trial.* **J Cardiovasc Pharmacol** 52(5):393-402,2008. | | | **3** | |
| 26 – ***The reduction in post-prandial hyperglycemia with acarbose was associated with a 49% reduction in the relative risk (RR) of developing cardiovascular events (HR 0.51). Among cardiovascular events, the most important reduction was in the risk of myocardial infarction (HR 0.09). Acarbose also caused a 34% reduction in the incidence of new cases of hypertension.*** | | Chiasson JL et al. *Acarbose Treatment and the Risk of Cardiovascular Disease and Hypertension in Patients With Impaired Glucose Tolerance.* **JAMA 2003,**290:486-494. | | | **3** | |
| 27 – ***In a review of 30 clinical studies, it was not possible to find evidence whether alpha-glucosidase inhibitors influence mortality or morbidity in T2DM patients. On the other hand, they show a significant effect on glycemic control and on insulin levels, but no effect statistically significant on lipids or on body weight.*** | | Van de Laar FA et al. *Alpha-Glucosidase Inhibitors for Type 2 Diabetes Mellitus*. **Cochrane Database Syst Rev** 2005, 18;(2):CD003639. | | | **3** | |
| 28 – ***Therapy with orlistat promotes clinically significant weight loss with improvement in glycemic control and in risk factors for cardiovascular disease in obese or overweight T2DM patients with poor metabolic control despite insulin therapy.*** | | Kellev DE et al. *Clinical Efficacy of Orlistat Therapy in Overweight and Obese Patients with Insulin-Treated Type 2 Diabetes: a 1-Year Randomized Controlled Trial*. D**iabetes Care 2003,** 26(3):971.  Berne C et al. *A Randomized Study of Orlistat in Combination with a Weight Management Programme in Obese Patients with Type 2 Diabetes Treated with Metformin*. **Diabet Med 2005,**22(5):612-8. | | | **3** | |
| 29 – ***Fluoxetine, orlistat and sibutramine may promote statistically significant weight loss during a period of 12-57 weeks. However, weight loss magnitude is modest and long-term health benefits are still questionable.*** | | Norris SL et al. *Pharmacotherapy for Weight Loss in Adults with Type 2 Diabetes Mellitus*. **Cochrane Database Syst Rev** 2005 Jan 25;(1):CD004096. | | | **4** | |
| 30 – ***The treatment with topiramate promotes significant weight loss and significant improvement in A1C and blood pressure levels in obese T2DM patients treated with diet and exercise or using metformin. However, psychiatric adverse events and CNS events caused by topiramate make it unsuitable to treat obesity and diabetes.*** | | Rosenstock J et al. *A Randomized, Double-Blind, Placebo-Controlled, Multicenter Study to Assess the Efficacy and Safety of Topiramate Controlled Release in the Treatment of Obese Type* *2 Diabetic Patients*. **Diabetes Care** 2007,30(6):1480-6.  Khanna V et al. *Topiramate and Type 2 Diabetes: na Old Wine in a New Bottle*. **Expert Opin Ther Targets 2008,**12(1):81-90. | | | **4** | |
